# Perceptions, Barriers and Pathways Forward: A Scoping Review of CALD Participation in Australian Clinical Trials

**DOI:** 10.1111/hex.70804

**Published:** 2026-09-04

**Authors:** Thy Vuong, Manju Daniel, Krishna Lambert

**Affiliations:** ^1^ Cardiology Department Canberra Health Services Garran Australian Capital Territory Australia; ^2^ Rush University Chicago Illinois USA; ^3^ University of Canberra Bruce Australian Capital Territory Australia

## Abstract

**Background:**

Equitable participation in clinical trials is essential for ensuring that research evidence is scientifically robust, ethically just and representative of the populations it is intended to serve. In Australia, culturally and linguistically diverse (CALD) populations remain underrepresented in clinical trials despite increasing national policy attention to equity and inclusion. This review synthesized empirical evidence and Australian policy and practice documents to examine factors influencing CALD participation in clinical trials.

**Objectives:**

To synthesize evidence on CALD attitudes, perceptions and factors influencing clinical trial participation in Australia; examine reported barriers and facilitators; describe how CALD populations have been conceptualized across the literature; and identify implications for research, practice and policy.

**Methods:**

A scoping review was conducted in accordance with Joanna Briggs Institute guidance and reported using PRISMA‑ScR. Five electronic databases were searched for English‑language primary studies published between 2006 and June 2025. Eligible studies examined CALD adults' perceptions, experiences or participation in Australian clinical trials. A targeted synthesis of Australian grey literature was undertaken to contextualize empirical findings within the national policy and practice landscape. Data were charted and synthesized narratively.

**Results:**

Eight empirical studies and seven Australian policy and practice documents were included. Five themes emerged from the empirical literature: (1) willingness to participate exists but opportunity is structurally constrained; (2) language and health literacy are pervasive barriers; (3) cultural values, family involvement and trust shape participation decision‐making; (4) system‐level practices limit equity monitoring and inclusion; and (5) inconsistent implementation of feasible facilitators. Policy documents increasingly recognized these same structural determinants and proposed system‐level strategies to improve equitable participation; however, nationally consistent implementation and accountability mechanisms remain limited.

**Conclusion:**

Clinical trial participation among CALD populations should be understood as a dynamic, multi‐level decision‐making process rather than a single recruitment or informed consent event. Although Australia's policy landscape increasingly recognizes the structural determinants of equitable participation, implementation remains fragmented. Future efforts should prioritize culturally responsive, evidence‐informed and measurable approaches that support equitable participation throughout the clinical trial pathway.

**Patient or Public Contribution:**

This scoping review synthesized published literature and did not involve the direct collection of data from patients or members of the public. The analysis, interpretation and framing of the findings were directly informed by patient and community perspectives reported in the included studies, many of which involved CALD consumers, carers and community members. The review was designed to amplify these perspectives and identify system‑level changes to improve equity in clinical trial participation.

## 1 Introduction

Clinical trials are internationally recognized as the cornerstone of evidence‐based healthcare, generating the evidence that informs the safety, efficacy and implementation of new interventions while informing clinical practice, health policy and healthcare delivery [1, 2, 3, 4]. The validity and applicability of clinical trial findings depend on trial populations that are representative of the communities who will ultimately receive these interventions [5, 6]. The exclusion or systematic underrepresentation of population groups compromises the external validity and generalizability of research findings, restricts the applicability of evidence to real‐world populations and may perpetuate inequities in access to emerging therapies and evidence‐based care [5, 6]. Beyond scientific considerations, equitable participation in clinical trials is both an ethical imperative grounded in the principles of justice, respect, beneficence and research merit and integrity articulated within the National Statement on Ethical Conduct in Human Research and internationally recognized ethical frameworks [7, 8, 9]. Achieving equitable participation therefore requires intentional and inclusive strategies to ensure all populations have meaningful opportunities to learn about, consider and participate in clinical research.

Australia is one of the most culturally diverse nations globally, with approximately one‐third of (29.3%) Australian residents born overseas, and around half (51.5%) have at least one parent born outside of Australia, and more than 300 languages are spoken nationally [10, 11]. As Australia's population continues to diversify, ensuring equitable opportunities to participate in clinical trials has become increasingly important to ensure that research evidence reflects the communities it is intended to serve. Within Australian health policy and research, these communities are commonly described using the umbrella term “culturally and linguistically diverse” (CALD), a broad construct that captures dimensions of diversity extending beyond ethnicity or country of birth alone.

Despite its widespread use, the conceptualization of CALD remains inconsistent throughout literature. Rather than representing a single demographic characteristic, CALD is a multidimensional construct that may be described using combinations of country of birth, parental country of birth, language spoken at home, English proficiency, Indigenous status, ancestry, religious affiliation and year of arrival [12]. Consequently, studies frequently conceptualize CALD using different combinations of these indicators, creating challenges for comparing findings, monitoring equity and synthesizing evidence across studies [13, 14]. Accordingly, examining how CALD has been conceptualized is an important consideration when interpreting the Australian clinical trial literature; therefore, it forms an important component of this review.

Recent systematic and scoping reviews have advanced understanding of equitable participation in clinical trials among CALD and minority populations globally. Collectively, these reviews have identified recurrent barriers to participation, including limited awareness of clinical trials, language barriers, low health literacy, culturally incongruent consent processes, mistrust, logistical constraints and clinician gatekeeping, while highlighting promising strategies such as multilingual study materials, interpreter services, culturally tailored recruitment, community partnerships and culturally competent research teams [15, 16, 17, 18, 19]. However, existing reviews have largely focused on specific disease settings, intervention strategies, recruitment approaches, or international populations, with limited attention to the unique Australian context in which CALD populations are conceptualized, identified and engaged in clinical research.

Within Australia, reviews have examined factors influencing overall participation in specific settings such as oncology clinical trials [19] and elderly CALD populations [6], while others zoomed into public involvement in clinical trials more broadly [20]. Despite these contributions, little synthesis has focused specifically on Australian CALD populations across the clinical trial pathway, and limited attention has been paid to how CALD populations have been conceptualized across studies. This is an important gap because substantial variation in the use of indicators such as country of birth, language spoken at home, English proficiency, ethnicity and migrant status may influence who is identified as CALD, complicating comparisons across studies and limiting efforts to monitor equity and representation [13, 14]. Furthermore, although Australia has introduced substantial policy, governance and ethical reforms aimed at promoting equitable clinical trial participation, including increased emphasis on consumer partnership, cultural responsiveness and health equity [13, 21, 22], there has been little synthesis examining how these policy priorities align with the experiences and determinants reported in the empirical literature. To our knowledge, no previous review has synthesized Australian evidence on CALD attitudes, perceptions, participation decision‐making, barriers and facilitators while simultaneously examining variation in CALD conceptualization and contextualizing these findings within Australia's contemporary clinical trial policy and practice landscape.

As Australia continues to strengthen its commitment to equitable clinical trial engagement, involvement and participation through national policy and governance initiatives, understanding how these priorities align with the experiences of CALD populations is increasingly important. A contemporary synthesis of the Australian evidence base is needed to inform culturally responsive research practices, support implementation of emerging policy priorities and identify areas where further action is required to promote equitable participation. Accordingly, this scoping review therefore aims to address the question: What is known about the attitudes, perceptions and factors influencing clinical trial participation among CALD populations in Australia?

The objectives of this review were to synthesize evidence on CALD attitudes, perceptions and factors influencing clinical trial participation in Australia; examine reported barriers and facilitators to participation; describe how CALD populations have been conceptualized across the Australian literature; and identify gaps and generate recommendations for future research, practice and policy.

## 2 Methods

### 2.1 Design

A scoping review was undertaken because the evidence relating to CALD populations and clinical trial participation in Australia is emerging, methodologically heterogeneous and with variation in how CALD populations are conceptualized. Consistent with Joanna Briggs Institute (JBI) guidance, scoping reviews are particularly appropriate for mapping the extent, range and characteristics of evidence, clarifying key concepts, examining how constructs have been operationalized and identifying knowledge gaps where systematic reviews of intervention effectiveness would be premature [23]. This review followed the JBI methodology for scoping reviews using the Population‐Concept‐Context (PCC) framework [23] and is reported in accordance with the Preferred Reporting Items for Systematic Reviews and Meta‐Analyses extension for Scoping Reviews (PRISMA‐ScR) [24]. The review protocol was prospectively registered with the Open Science Framework (OSF). The population of interest comprised CALD adults; the concept focused on attitudes, perceptions, barriers, facilitators and factors influencing clinical trial participation; and the context was Australian clinical trial and research settings.

### 2.2 Eligibility Criteria

Eligible studies were primary research conducted in Australia that examined CALD adults' perceptions, attitudes, experiences, decision‐making, barriers, facilitators or willingness to participate in clinical trials. Qualitative, quantitative and mixed‑methods designs were included. Studies published in English between 2006 and June 2025 were eligible. Studies focusing exclusively on Aboriginal and Torres Strait Islander peoples were excluded due to their distinct cultural, historical and governance contexts requiring dedicated research frameworks [25]. Studies conducted outside Australia, those unrelated to clinical research participation, editorials, commentaries, conference abstracts, protocols and secondary evidence (e.g., literature reviews and secondary analysis) were also excluded. Temporal limits reflected substantial reform and expansion of the Australian clinical trials ecosystem since 2006, as documented in national clinical trial infrastructure reviews [21, 26].

To contextualize the empirical findings within Australia's evolving clinical trial policy and practice landscape, a targeted search of Australian grey literature was undertaken during manuscript revision. Sources included national policy documents, government reports, clinical trial guidance documents and reports from peak professional organizations relevant to equitable clinical trial engagement, involvement and participation among CALD populations. Searches were conducted using Google and targeted searches of organizational websites, including the Australian Clinical Trials Alliance, National Health and Medical Research Council, Australian Commission on Safety and Quality in Health Care, Australian Institute of Health and Welfare, and the Australian Government Department of Health. Documents were eligible if they addressed Australian clinical trial policy, governance, workforce capability or strategies relevant to equity in clinical trials. The grey literature search was purposive rather than exhaustive.

### 2.3 Information Sources and Search Strategy

A comprehensive literature search was conducted across PubMed, Embase, CINAHL, Scopus and PyscINFO. These databases were selected as they collectively index health sciences, clinical research and social science domains central to understanding CALD representation. To maximize search sensitivity, database searches were supplemented by Google Scholar and manual screening of reference lists from included studies and relevant reviews. Search strategies combined controlled vocabulary (e.g., Medical Subject Headings [MeSH]) with relevant free‐text terms such as: “culturally and linguistically diverse,” “CALD,” “ethnic minorities,” “migrants,” “clinical trials,” “research participation,” “Australia,” “perception,” “attitudes,” “barriers” and “facilitators.” Boolean operators (AND/OR) and truncation symbols were used to improve sensitivity and specificity of results. Searches were limited to English‐language studies from 2006 to 24 June 2025. Sources met the eligibility criteria were stored in Zotero citation management software. A targeted synthesis of Australian grey literature was undertaken to contextualize empirical findings within the national policy and practice landscape.

### 2.4 Study Selection

Following duplicate removal, two reviewers independently screened titles and abstracts against the eligibility criteria. Full‐text articles were subsequently assessed independently by both reviewers. Disagreements were resolved through discussion and consensus. The study selection process is presented using the PRISMA‐ScR flow diagram.

### 2.5 Data Charting

Data were extracted using a JBI‑informed charting template. Extracted variables included study characteristics (author, year, design, setting, population, sample size), participant characteristics, methods, aims, and key findings relating to attitudes, perceptions, decision‐making, barriers and facilitators. Additionally, data were extracted regarding how each study conceptualized CALD (e.g., country of birth, language spoken at home, English proficiency, ancestry or other indicators).

### 2.6 Data Synthesis

Given the methodological heterogeneity of the included studies, statistical pooling was neither appropriate nor feasible. Findings were synthesized narratively guided by established approaches to narrative synthesis [27]. Thematic analysis identified recurrent patterns relating to perceptions of clinical trials, barriers and facilitators to participation, and broader system‐level influences across study designs and clinical contexts. Grey literature was synthesized separately from empirical studies and is presented descriptively to provide policy and practice context. As these documents were used solely to contextualize the empirical findings rather than contribute participant‐level evidence, they were not included in the PRISMA‐ScR study selection process or flow diagram.

## 3 Results

### 3.1 Overview of Included Studies

**Figure 1:**
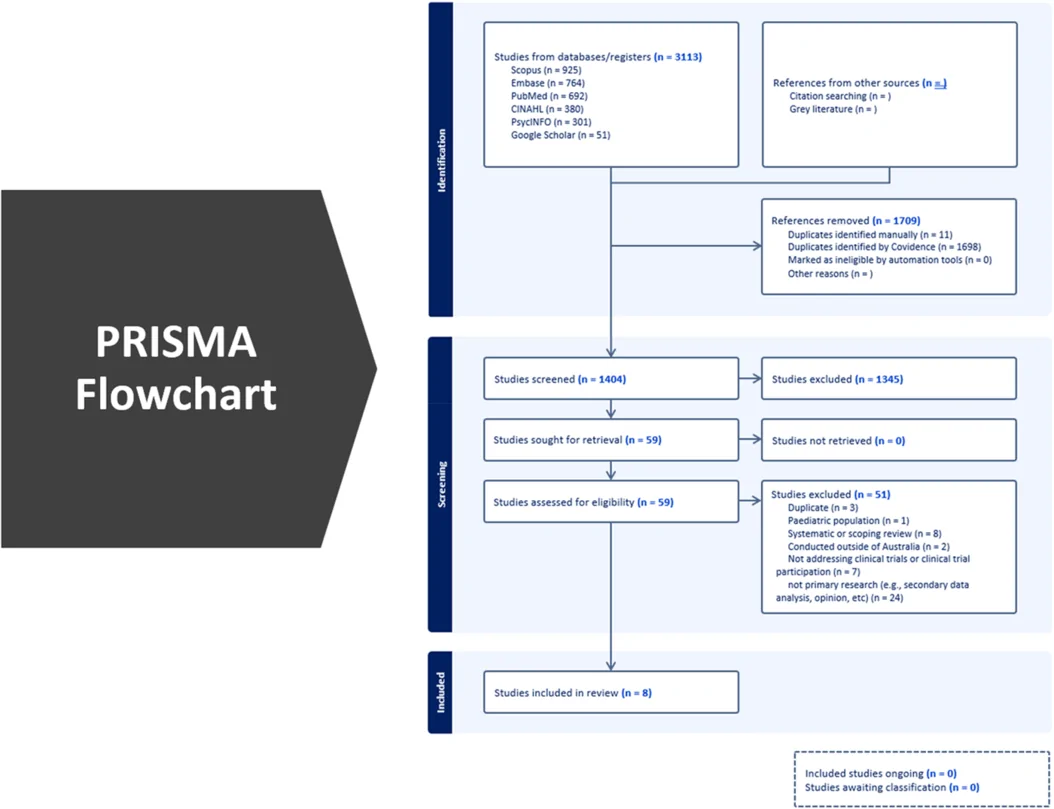
PRISMA Flowchart.

Eight empirical studies published between 2006 and 2025 met inclusion criteria (Figure 1; Table 1), comprising qualitative (*n* = 2), quantitative descriptive and cohort (*n* = 2) and mixed‐methods designs (*n* = 4) [28, 29, 30, 31, 32, 33, 34, 35]. Studies were concentrated in oncology, ageing and health services research, with minimal representation from other high‐burden specialties [21, 36]. Most were conducted in metropolitan centres; rural and remote settings were underrepresented. In addition, seven Australian policy and practice documents were identified through the targeted grey literature search and are summarized separately in Supporting Information S1: Table S1.

**Table 1:** Study characteristics.

Author(s) (year) [Ref.]	Design and setting	Population (*n*)	Aims	Measures/Approach	CALD definition
Brijnath et al. (2024) [28]	Qualitative; 16 focus groups; six Australian capitals + one rural town	Adults from 21 ethnically diverse communities (*n* = 158). Aged 18–85, 49% women; participated in groups; speaking Tamil, Greek, Punjabi, Italian, Mandarin, Cantonese, Karin, Vietnamese, Nepalese, and Arabic; or English‐language groups (comprising Fijian, Filipino, African and two multicultural groups).	Explore ethnically diverse communities' perspectives on improving ethnic diversity in trials (recruitment, consent, data collection, retention)	Focus‑group guide; NVivo thematic analysis (realist framework)	Ethnicity defined by shared culture, history, language
Feldman et al. (2008) [29]	Mixed‑methods (process evaluation within quantitative study); three LGAs, Melbourne	Older adults (Recruited: *n* = 386; Interviewed: *n* = 341): ≥ 65 years from seven cultural groups (Vietnamese, Greek, Italian, Maltese, Macedonian, Croatian, Anglo‐Celtic)	Methodological reflection on recruiting/retaining older adults from seven cultural groups in a study of physical activity, built environment, leisure satisfaction.	Structured questionnaire (1‑h) interviews via bilingual interviewers; translated materials/consent; recruitment logs (council versus interviewer/key informant); attrition reasons; coordinator field notes and meeting minutes.	Self‐identified cultural group; language other than English
Hyatt et al. (2022) [30]	Mixed‑methods pilot RCT; two oncology services, Melbourne	Migrant oncology patients using interpreters (Arabic/Greek/Mandarin/Cantonese) (*n* = 47)	Pilot feasibility of methods (QPLs + recorded consultations) to include migrant oncology patients; test feasibility for future RCT of INFORM (translated QPLs + consultation recordings).	Feasibility metrics (recruitment/attrition/fidelity/cancellations); PROMs (PSCCS, SCNS‑SF‑R, STAI‑6; info recall via SSI); NAATI translations; bilingual RA training/logs; detailed time and costing.	Interpreter‑requiring migrant groups
Nindra et al. (2025) [31]	Prospective cohort (quantitative); two Phase‑I sites, Sydney	Early‑phase oncology trial participants (*n* = 114). Median age: 63 years (range = 25–83 years), predominantly female (52%). No LGBTIA+. 30% culturally diverse, 25% linguistically diverse. 1 Indigenous Australian; CALD, LGBTQIA+, Indigenous	Prospectively describe diversity (social, cultural, linguistic, economic, sexual, regional) among early‑phase trial participants; benchmark versus population; (secondary aims include HRQoL and clinical outcomes).	Baseline survey (cultural/linguistic status; sexual orientation; parental country of birth; preferred language; ABS ENGLP A–E; distance); ABS IRSAD (postcode); ECOG, cancer type; trial phase and modality; EORTC QLQ‑C30 cycles 1–6.	Born in non‐English‐speaking country or parent born overseas where English is not predominant
Pal et al. (2023) [32]	National online survey (quantitative + qualitative) of workforce	Clinicians/research staff (*n* = 91) working with CALD cancer patients	Identify barriers and solutions to improve CALD recruitment to Australian cancer trials from the trials workforce perspective.	REDCap 15‑item survey (closed/open items); descriptive analysis; NVivo v12 thematic analysis; national distribution via COSA/MOGA/cooperative groups.	Australian term CALD (country of birth, country of birth of parents, main language other than English spoken at home, proficiency in spoken English, preferred language and indigenous status).
Perrins et al. (2021)[33]	Mixed‑methods evaluation; regional Queensland	CALD health consumers in a literacy programme (*n* = 40)	Evaluate recruitment and retention methods for hard‑to‑reach regional CALD participants pre‑ versus during COVID; within a health‑literacy profiling and education intervention.	HLQ (21‑item Likert; pre/post); semi‑structured interviews aligned to HLQ domains; observational logs and participant feedback (survey/interview); phase‑wise counts across five stages	Migrant background, language other than English
Smith et al. (2020) [34]	Cross‑sectional survey (quantitative); South Western Sydney	Vietnamese‐Australian (*n* = 50) versus Anglo‐Australian cancer patients (*n* = 100)	Compare trial knowledge/attitudes in Vietnamese versus Anglo cancer patients; identify correlates; test whether knowledge/attitudes predict future participation.	Knowledge and Attitudinal Barrier Survey (47 items; *α* = 0.71/0.93); Information Styles (1‑item); Control Preferences (1‑item); Health Literacy Screening (3‑item; *α* = 0.73); past/future participation items; demographics; regression analyses.	ABS definition: born in non‐English‐speaking countries
Woodward‐Kron et al. (2016) [35]	Qualitative interviews and focus groups; Melbourne	Older Italian‑Australians (low literacy) (*n* = 21); family carers (*n* = 10); HREC admins (*n* = 4); researchers (*n* = 4)	Elicit older Italian CALD participants’ and stakeholders’ experiences on medical care and research; explore acceptability of multimedia/e‑consent	Semi‑structured interviews and focus groups (4 tailored guides; Italian translation); thematic analysis	Non‐English‐speaking migrant background

### 3.2 Conceptualization of CALD

Conceptual definitions of CALD populations varied substantially across studies. Some utilized Australian Bureau of Statistics (ABS) indicators such as country of birth, language spoken at home and English proficiency [29, 32, 34], whereas others relied on self‐identified ethnicity or inferred cultural background (Table 1) [28, 30, 33, 35]. Few studies explicitly justified their conceptual definitions, and no standardized approach was observed across the literature. The lack of consistent conceptual definitions limited comparability across studies and complicated interpretation of CALD representation within the Australian clinical trial literature.

### 3.3 Thematic Findings

Despite methodological and contextual heterogeneity, the empirical evidence (Table 2) consistently converged on five core themes shaping CALD participation in Australian clinical trials.

### 3.4 Theme 1: Willingness to Participate Exists, but Opportunity Is Structurally Constrained

Across patient‐ and community‐facing studies, CALD participants generally expressed openness to, and interest in, participating in clinical trials. However, this willingness co‐existed with limited exposure to trial opportunities. Participants commonly reported not being informed about trials, not being invited to participate, or perceiving that trials were implicitly “not for people like us” [28, 34]. Quantitative findings suggested that disparities in clinical trial participation between CALD and non‐CALD populations were not solely driven by attitudes toward research, but also by limited participation opportunities, including lower trial awareness, language‐related barriers, and reduced access to referral and recruitment pathways [34]. Qualitative findings corroborated these patterns, highlighting the central role of clinician‐mediated recruitment pathways and the influence of implicit assumptions regarding language proficiency, comprehension and presumed willingness to participate [28, 29, 31, 35]. Collectively, these findings consistently identified limited trial awareness, infrequent invitations and clinician‐mediated recruitment pathways as factors associated with lower CALD participation across multiple clinical contexts.

### 3.5 Theme 2: Language and Health Literacy as Pervasive and Modifiable Barriers

Language proficiency and health literacy emerged as the most consistently reported barriers to CALD participation across all included studies. Participants described difficulty understanding trial information due to complex medical terminology, lengthy and technical consent documents, and limited access to translated or plain‐language materials [31, 32, 35]. From the workforce perspective, clinicians and research staff identified inadequate access to professional interpreters, insufficient funding for translation services and lack of culturally appropriate informational resources as key operational constraints [29]. National workforce survey data further indicated that communication‐related barriers, such as low English proficiency, low health literacy and absence of simplified materials, were among the most frequently cited obstacles to inclusive trial recruitment in Australia [29]. These barriers were evident across metropolitan and regional settings, with additional logistical constraints (e.g., transport, scheduling, childcare) compounding communication challenges, particularly outside major urban centres [33].

### 3.6 Theme 3: Cultural Values, Family Involvement and Trust Shape Participation Decision‐Making

Decision‐making regarding trial participation was embedded within relational and cultural contexts rather than being solely individualistic. CALD participants frequently described family involvement as integral to understanding trial information, deliberating risks and benefits, and arriving at participation decisions, particularly among older adults and individuals with limited English proficiency [35]. Cultural norms, which include deference to medical authority, reluctance to question clinicians, and sensitivities surrounding illness, randomization, and biological sample collection, shaped how trial information was interpreted and whether participation was perceived as appropriate [28]. Some participants reported “getting by” in clinical encounters despite limited comprehension, raising concerns regarding the adequacy of informed consent processes and the ethical robustness of participation in linguistically discordant settings [35]. Trust in clinicians and institutions emerged as a critical facilitator of engagement. Where trust was fostered through continuity of care, culturally responsive communication and community‐linked engagement, participants reported greater comfort with trial participation [31, 32, 33, 35]. Conversely, uncertainty regarding data use, sample storage and institutional motives diminished willingness to engage [28].

### 3.7 Theme 4: System‐Level Practices Limit Equity Monitoring and Inclusion

Several included studies identified system‐level constraints that limit equitable CALD inclusion in Australian clinical trials. These included exclusionary eligibility criteria (e.g., English proficiency requirements), limited resourcing for inclusive recruitment strategies, and the absence of standardized collection of ethnicity, language and interpreter‐use data within trial workflows and reporting systems [29]. Prospective cohort and workforce survey data indicated that most Australian trial sites do not routinely collect CALD‐related demographic variables, constraining the capacity to monitor representation, identify inequities or evaluate the impact of equity‐focused interventions [29, 30]. Where diversity data were available, CALD participation remained variable and uneven across trial phases and disease areas.

### 3.8 Theme 5: Facilitators Are Feasible but Inconsistently Implemented

Across the included studies, facilitators to CALD participation were consistently identified and largely feasible within existing Australian health service infrastructures. These included access to professional interpreters and bilingual research staff, multilingual and audiovisual information materials, patient navigation roles, community partnerships, family‐inclusive consent processes, and flexible trial logistics [28, 31, 32]. Intervention‐focused studies demonstrated that once workflows were established, such strategies could be operationalized with manageable resource implications to increase CALD participation [32]. However, implementation was inconsistent and often contingent on local champions, short‐term project funding or individual clinician commitment rather than embedded organizational policy or system‐level infrastructure [29].

### 3.9 Australian Policy and Practice Context

A targeted review identified seven Australian policy and practice documents published between 2020 and 2026 that were relevant to equitable clinical trial engagement, involvement and participation among CALD populations (Supporting Information S1: Table S1). These comprised an environmental scan, national recommendations, governance and operational guidance, national health service standards, a national health and medical research strategy, and Australia's national ethical guidance for human research [2, 7, 13, 22, 37, 38, 39]. Collectively, the documents reflect an evolution in Australia's policy landscape from identifying inequities in clinical trial participation [37] toward establishing governance expectations, operational guidance, ethical principles and strategic priorities to support more equitable and culturally responsive clinical trial ecosystems [2, 7, 13, 22, 38].

Across policy and practice documents, implementation priorities consistently focused on strengthening organizational capability to support more equitable clinical trial participation among CALD populations [2, 13]. Recurring recommendations included developing community partnerships and consumer engagement, improving community clinical trial literacy, enhancing workforce capability through culturally responsive training, providing multilingual participant resources and interpreter services, routinely collecting CALD demographic characteristics and embedding consumer involvement throughout the research lifecycle [13, 37]. These priorities were further supported by national governance and operational guidance, which emphasized participant‐centred communication, health literacy, standardized trial processes and quality management systems to support more inclusive clinical trial delivery [22, 38, 39]. Similarly, the grey literature consistently positioned equitable clinical trial engagement, involvement, and participation as an organizational and system‐level responsibility, with emphasis on strengthening governance, workforce capability, consumer partnership, culturally responsive communication and continuous quality improvement across the clinical trial lifecycle.

## 4 Discussion

This scoping review synthesized empirical evidence alongside contemporary Australian policy and practice documents to examine factors influencing clinical trial participation among CALD populations in Australia. The empirical findings identified in this review collectively point to CALD participation in clinical trials as a multi‐level decision‐making process that begins long before informed consent encounters. Opportunity structures (Theme 1) shape whether individuals are aware of clinical research and are invited to consider participation. Once opportunities are presented, decision‐making is further influenced by language proficiency, clinical trial knowledge and health literacy (Theme 2), which impact comprehension of complex trial information. Additionally, these factors intersect with relational dynamics (Theme 3), where trust in clinicians and researchers, culturally responsive communication, and family involvement shape how risks and benefits are interpreted and participation decisions are made [28, 33, 35].

**Figure 2:**
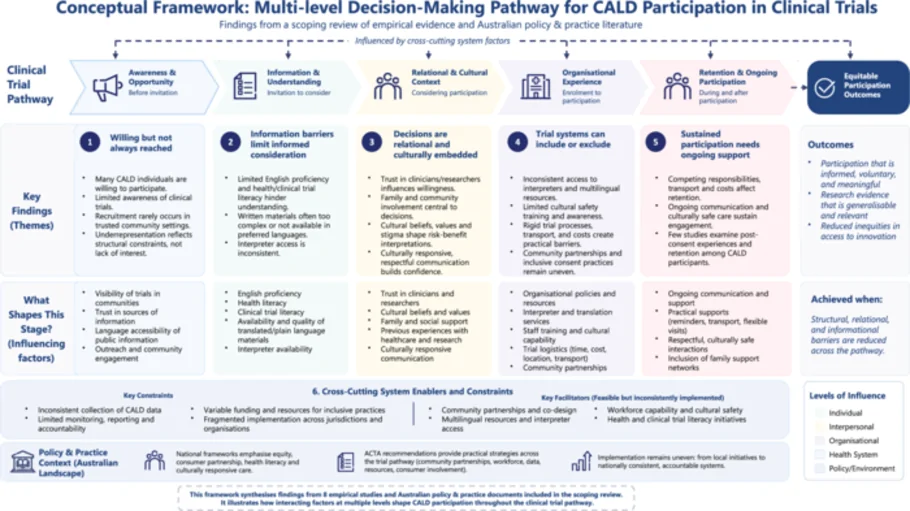
Multilevel decision‐making pathway for CALD participation in clinical trials.

Beyond the participant level, organizational practices such as access to interpreters and multilingual resources, community partnerships in recruitment, family‐inclusive consent processes and flexible trial logistics influence whether equitable participation can be achieved consistently rather than on local champions or ad hoc resources [28, 29, 31, 32, 33]. At the system level, inconsistent capture of CALD‐related data and limited resourcing for inclusive practices (Theme 4) constrain both accountability and intervention, despite the feasibility of known facilitators (Theme 5) [29, 30]. Together, these findings shift the focus from viewing underrepresentation as a consequence of participant unwillingness towards recognizing participation as a dynamic process shaped by interacting individual, interpersonal, organizational and health system factors throughout the clinical trial pathway. These interacting influences are synthesized into a conceptual framework (Figure 2), which illustrates how modifiable determinants operate across successive stages of the clinical trial pathway and identifies potential points for intervention to support equitable participation among CALD populations.

Integrating the empirical findings with the Australian policy and practice literature provide important context. National governance and ethical frameworks emphasize equity, consumer partnership, health literacy and culturally responsive care, while Australian Clinical Trials Alliance (ACTA) extends these principles into practical recommendations, including community partnerships, clinical trial literacy, workforce capability, interpreter access, multilingual resources, consumer involvement and routine collection of CALD demographic data [2, 13, 22]. Importantly, these priorities closely mirror the determinants identified across the empirical studies, suggesting that Australia's policy landscape increasingly recognizes the same structural barriers influencing equitable participation. The grey literature also identifies that the implementation of strategies to address such barriers remain fragmented.

Current national guidance establishes broad principles for equitable research but does not prescribe consistent requirements for CALD recruitment, measurement or reporting [13]. Consequently, implementation continues to depend largely on local organizational capacity and jurisdictional initiatives, such as the Cancer Institute NSW multicultural clinical trial resources [40], rather than coordinated national infrastructure. Together, the empirical, policy and practice evidence suggests that Australia's principal challenge is embedding, resourcing and evaluating culturally responsive practices across the clinical trial system.

This implementation gap has important ethical as well as scientific implications. Respect for persons, justice, beneficence and research merit require more than equitable eligibility criteria or the provision of written information; they require that prospective participants are adequately supported to understand, deliberate and voluntarily decide whether participation aligns with their values and circumstances [8, 41]. When communication barriers, limited health literacy support and culturally incongruent research processes persist, informed consent may not fully achieve its ethical purpose. Likewise, when modifiable organizational barriers systematically reduce opportunities for CALD communities to participate, the principle of justice is challenged. Framing equitable participation as a shared organizational and system‐level responsibility therefore strengthens both the scientific validity and ethical integrity of Australian clinical trials.

The findings of this review also have implications for research, practice and policy. At the participant level, strategies that strengthen clinical trial literacy, support culturally responsive communication and acknowledge the influence of trust and family involvement may better support informed participation decision‐making. For researchers and research teams, recruitment should extend beyond translation of study materials to incorporate meaningful community partnerships, appropriate use of interpreters, consumer and carer involvement, and culturally responsive engagement throughout the research lifecycle. Organizationally, sustainable improvements will require investment in workforce capability, multilingual resources, routine collection of CALD demographic characteristics and governance processes that embed equity within routine clinical trial conduct. Nationally, greater consistency in defining, measuring and reporting CALD participation, alongside resources and funding to support implementation of ACTA's recommendations, could strengthen accountability and facilitate evaluation of progress towards more equitable clinical trial systems.

This review also identifies important priorities for future research. Existing Australian studies have largely described barriers and facilitators to participation, with comparatively little attention to how potentially modifiable determinants interact to influence participation decision‐making among CALD populations (Table 2). The conceptual framework developed from this synthesis (Figure 2) suggests that factors such as clinical trial literacy, trust in medical researchers, participation‐related beliefs, family and social support, and opportunity for engagement may influence participation decisions across different stages of the clinical trial pathway. Future research should move beyond describing inequities towards examining these relationships and evaluating culturally responsive interventions capable of improving equitable clinical trial engagement, involvement and participation.

This review has several strengths. It is, to our knowledge, the first Australian scoping review to synthesize empirical evidence alongside national policy and practice documents relating to clinical trial participation among CALD populations. By explicitly examining how CALD populations were conceptualized across literature, the review also addresses an important methodological gap identified by previous reviews. The review followed established JBI methodology, adhered to PRISMA‐ScR reporting guidance and incorporated contemporary Australian policy documents to contextualize the empirical findings.

Nevertheless, several limitations should be acknowledged. Although only eight empirical studies met the eligibility criteria, we believe this reflects the current state of the Australian literature rather than inadequate search sensitivity. The limited number of available studies, together with their methodological heterogeneity and inconsistent conceptualization of CALD, constrained the breadth of evidence that could be synthesized and limited comparisons across clinical contexts. Although the targeted grey literature synthesis enhanced interpretation of the empirical findings within the Australian policy context, the absence of a predefined, systematic search strategy and inclusion of additional relevant policy and practice documents may have limited the comprehensiveness of the review findings.

## 5 Conclusion

This review demonstrates that equitable clinical trial participation among CALD populations should be understood as a dynamic, multi‐level decision‐making process rather than a single recruitment or informed consent event. By integrating empirical evidence with Australia's evolving policy and practice landscape, this review highlights growing consensus regarding the structural determinants of equitable participation while identifying a persistent gap between policy aspirations and routine implementation. Closing this implementation gap will require coordinated action across researchers, health services, policymakers, sponsors and communities to embed culturally responsive, evidence‐informed and measurable approaches throughout the clinical trial pathway. Future research should build on this foundation by examining modifiable determinants of participation decision‐making and evaluating strategies that improve equitable clinical trial engagement, involvement and participation. Ultimately, ensuring that all Australians have meaningful opportunities to participate in clinical research is fundamental to generating evidence that is not only scientifically robust but also ethically just and representative of the populations it is intended to serve.

**Table 2:** Perceptions, barriers and facilitators.

Author(s), year [Ref.]	Population (*n*)	Key attitudes/Perceptions	Barriers	Facilitators/Solutions
Brijnath et al. (2024) [28]	Ethnically diverse adults (*n* = 158); *N* = 158. Aged 18–85, 49% women; participated in groups; speaking Tamil, Greek, Punjabi, Italian, Mandarin, Cantonese, Karin, Vietnamese, Nepalese, and Arabic; or English‐language groups (comprising Fijian, Filipino, African and two multicultural groups).	Support for research but lack of invitations; concerns about taboo topics, physical specimens and sensitive cultural questions	Under‑invitation; heavy written load; taboo/specimen concerns; fear of discrimination; transport	Community partners; translated and concise materials; AV consent; rapport; transparency on samples/confidentiality; flexible scheduling.
Feldman et al. (2008) [29]	Older adults (recruited: *n* = 386; interviewed: *n* = 341): ≥ 65 years from seven cultural groups (Vietnamese, Greek, Italian, Maltese, Macedonian, Croatian, Anglo‐Celtic)	Trust and relationships critical; skepticism toward research; cultural norms influence participation	Language/literacy; mistrust; fear of signing; limited council staff time/resources	Bilingual interviewers; key informants; flexible recruitment; translated materials
Hyatt et al. (2022) [30]	Migrant oncology patients using interpreters (Arabic/Greek/Mandarin/Cantonese) (*n* = 47)	Migrant patients hesitant; family involvement critical	Language/literacy; unfamiliarity; stigma; family gatekeeping; time	QPLs +recorded consults; bilingual RAs; consumer advocates; interpreter integration; feasible costs
Nindra et al. (2025) [31]	Early‑phase oncology trial participants (*n* = 114). Median age: 63 years (range = 25–83 years), predominantly female (52%). No LGBTIA+. 30% culturally diverse, 25% linguistically diverse. 1 Indigenous Australian; CALD, LGBTQIA+, Indigenous	CALD and linguistically diverse patients represented (~ 30%), but Indigenous and LGBTQIA+ severely underrepresented in these early phase clinical trials	Not barrier‑focused; signals under‑representation of Indigenous and LGBTQIA+	Recommend multilingual materials; navigators; decentralized/tele‑trial options; standardized diversity capture
Pal et al. (2023) [32]	Clinicians/research staff (*n* = 91) working with CALD cancer patients	CALD patients underrepresented; communication barriers dominate	Low English/health literacy; lack of translations; exclusionary protocols; funding/time	Patient navigators; multilingual pamphlets/videos; site‑level process changes; routine diversity data collection
Perrins et al. (2021) [33]	CALD health consumers in a literacy program (*n* = 40)	Fear of research, need for trust, preference for informal sessions	Social isolation; language; low awareness; transport/childcare	Gatekeepers; smaller homogenous groups; flexible times; digital platforms
Smith et al. (2020) [34]	Vietnamese‐Australian (*n* = 50) versus Anglo‐Australian cancer patients (*n* = 100)	Vietnamese participants had more negative attitudes; similar knowledge levels	Cost misconceptions; negative attitudes; reluctance to ask; concerns re: randomization	Tailored education; clinician‑initiated offers; family engagement; navigation
Woodward‐Kron et al. (2016) [35],	Older Italian–Australians (low literacy) (*n* = 21); family carers (*n* = 10); HREC admins (*n* = 4); researchers (*n* = 4)	CALD participants feel like they were “getting by” in medical interactions as well as experiences of trial participation and recruitment, and excluded (because of low English proficiency and “too many health issues”); family plays key role; respect for doctors	Interpreter/translation resource constraints; complex consent; mistrust; exclusion of low‑English speakers	Family‑inclusive strategies; ethics education; alternative consent/AV tools; prototype bilingual multimedia resource

## Author Contributions

**Thy Vuong:** conceptualization, investigation, writing – original draft, methodology, validation, visualization, writing – review and editing, software, formal analysis, project administration, data curation, supervision, resources. **Manju Daniel:** methodology, data curation, writing – review and editing, validation, supervision. **Krishna Lambert:** methodology, writing – review and editing, supervision.

## Funding

The authors have nothing to report.

## Ethics Statement

The authors have nothing to report.

## Conflicts of Interest

The authors declare no conflicts of interest.

## Supporting information


**Table S1:** Australian Grey Literature of Policy and Practice Recommendations on CALD Engagement in Clinical Trials.

## Data Availability

The data that support the findings of this study are available in the supplementary material of this article.

## Appendix A Search Strategies

Generic Search:

(“CALD” OR “cultur* and linguistic* divers*” OR “ethnic* divers*” OR “language divers*” OR “linguist* divers*” OR “cultur* divers*” OR “migrant group*” OR “ethnic* group*”)

AND (perception* OR perceive* OR perspective* OR interest* OR aware* OR barrier* OR facilitat* OR access* OR resist* OR willing* OR participat* OR attitude* OR knowledge)

AND (“Clinical Study” [publication type] OR “clinical stud*” OR “clinical trial*” OR “Comparative Study” [publication type] OR “Evaluation Studies” [publication type] OR “Multicenter Study” [publication type] OR “Support of Research” [publication type] OR blind* OR cohort OR double‐blind* OR mask* OR double‐mask* OR random* OR placebo* OR retrospect* OR prospective* OR study OR studies OR trial OR trials OR quasi‐randomi* OR quasi‐experi*)

AND (australia*))

PubMed: 692 Results

(“CALD” [tiab] OR “cultur* and linguistic* divers*” [tiab] OR “ethnic* divers*” [ti] OR “language divers*” [ti] OR “linguist* divers*” [ti] OR “cultur* divers*” [ti] OR “migrant group*” [ti] OR “ethnic* group*” [ti])

AND (perception* [tiab] OR perceive* [tiab] OR perspective*[Title/Abstract] OR interest*[Title/Abstract] OR aware*[Title/Abstract] OR barrier*[Title/Abstract] OR facilitat*[Title/Abstract] OR access*[Title/Abstract] OR resist*[Title/Abstract] OR willing*[Title/Abstract] OR participat* [Title/Abstract] OR attitude* [tiab] OR knowledge [tiab] OR “Cultural Competency”[MAJR])

AND (“Clinical Study”[PT] OR “clinical stud*” [tiab] OR “clinical trial*” [tiab] OR “Comparative Study”[PT] OR “Evaluation Studies”[PT] OR “Multicenter Study”[PT] OR “Epidemiologic Studies”[Mesh] OR “Support of Research”[PT] OR blind*[tiab] OR cohort [tiab] OR double‐blind* [tiab] OR mask* [tiab] OR double‐mask*[tiab] OR random* [tiab] OR placebo* [tiab] OR retrospect* [tiab] OR prospective* [tiab] OR study [tiab] OR studies [tiab] OR trial [tiab] OR trials [tiab] OR quasi‐randomi* [tiab] OR quasi‐experi* [tiab] OR “Clinical Trials as Topic”[MeSH])

AND (australia*[Title/Abstract] OR “Australia”[Mesh] OR Australia[Affiliation])

Embase: 764 Results

(“cald”:ab,ti OR “cultur* and linguistic* divers*”:ab,ti OR “ethnic* divers*”:ti OR “language divers*”:ti OR “linguist* divers*”:ti OR “cultur* divers*”:ti OR “migrant group*”:ti OR “ethnic* group*”:ti) AND (“perception*”:ab,ti “ OR perceive*”:ab,ti OR “perspective*”:ab,ti OR “interest*”:ab,ti OR “aware*”:ab,ti OR “barrier*”:ab,ti OR “facilitat*”:ab,ti OR “access*”:ab,ti OR “resist*”:ab,ti OR “willing*”:ab,ti OR “participat*”:ab,ti OR “attitude*”:ab,ti OR “knowledge”:ab,ti OR “cultural competence”/exp/mj) AND (“clinical study”:it OR “clinical stud*”:ab,ti OR “clinical trial*”:ab,ti OR “comparative study”:it OR “evaluation studies”:it OR “multicenter study”:it OR “epidemiology”/de OR “support of research”:it OR “blind*”:ab,ti OR “cohort”:ab,ti OR “double‐blind*”:ab,ti OR “mask*”:ab,ti OR “double‐mask*":ab,ti OR “random*”:ab,ti OR “placebo*”:ab,ti OR “retrospect*”:ab,ti OR “prospective*”:ab,ti OR “study”:ab,ti OR “studies”:ab,ti OR “trial”:ab,ti OR “trials”:ab,ti OR “quasi‐randomi*":ab,ti OR “quasi‐experi*”:ab,ti OR “clinical trial (topic)”/de) AND (“australia*”:ab,ti OR “australia”/exp OR “australia”:ff,ad)

Scopus: 925 Results

(((TITLE‐ABS (“CALD” OR “cultur* and linguistic* divers*“)) OR (TITLE (“ethnic* divers*” OR “language divers*” OR “linguist* divers*” OR “cultur* divers*” OR “migrant group*” OR “ethnic* group*”))) AND (TITLE‐ABS (perception* OR perceive* OR perspective* OR interest* OR aware* OR barrier* OR facilitat* OR access* OR resist* OR willing* OR participat* OR attitude* OR knowledge)) AND (TITLE‐ABS (“clinical stud*“ OR “clinical trial*” OR blind* OR cohort OR double‐blind* OR mask* OR double‐mask* OR random* OR placebo* OR retrospect* OR prospective* OR study OR studies OR trial OR trials OR quasi‐randomi* OR quasi‐experi*))) AND ((AFFIL (australia*)) OR (AFFILCITY (australia*)) OR (TITLE‐ABS (australia*)))

CINAHL: 375 Results

(XB (“CALD” OR “cultur* and linguistic* divers*”) OR TI (“ethnic* divers*” OR “language divers*” OR “linguist* divers*” OR “cultur* divers*” OR “migrant group*” OR “ethnic* group*”)) AND (XB (perception* OR perceive* OR perspective* OR interest* OR aware* OR barrier* OR facilitat* OR access* OR resist* OR willing* OR participat* OR attitude* OR knowledge) OR MM “Cultural Competence”) AND (PT (“Clinical Study” OR “Comparative Study” OR “Evaluation Studies” OR “Multicenter Study” OR “Support of Research”) OR XB (“clinical stud*“ OR “clinical trial*“ OR blind* OR cohort OR double‐blind* OR mask* OR double‐mask* OR random* OR placebo* OR retrospect* OR prospective* OR study OR studies OR trial OR trials OR quasi‐randomi* OR quasi‐experi*) OR MH “Clinical Trials + “) AND (XB australia* OR AF australia* OR MH “Australia+“)

APA PsycInfo: 301 Results

(XB (“CALD” OR “cultur* and linguistic* divers*”) OR TI (“ethnic* divers*” OR “language divers*” OR “linguist* divers*” OR “cultur* divers*” OR “migrant group*” OR “ethnic* group*”)) AND (XB (perception* OR perceive* OR perspective* OR interest* OR aware* OR barrier* OR facilitat* OR access* OR resist* OR willing* OR participat* OR attitude* OR knowledge) OR DE “Cultural Competence”) AND (PT (“Clinical Study” OR “Comparative Study” OR “Evaluation Studies” OR “Multicenter Study” OR “Support of Research”) OR XB (“clinical stud*” OR “clinical trial*” OR blind* OR cohort OR double‐blind* OR mask* OR double‐mask* OR random* OR placebo* OR retrospect* OR prospective* OR study OR studies OR trial OR trials OR quasi‐randomi* OR quasi‐experi*) OR DE “Clinical Trials”) AND (XB australia* OR AF australia*)

Google Scholar:

A combination of keywords from the generic search was searched.
