# Supplementary material for: Perceptions, Barriers and Pathways Forward: A Scoping Review of CALD Participation in Australian Clinical Trials
Source: Health Expect. 2026 Sep 4;29(5):e70804. doi: 10.1111/hex.70804 (PMC13543165; doi:10.1111/hex.70804)
Supplement: Supplementary file 1 [file HEX-29-e70804-s001.docx]

**Supplemental Table 1S. Australian Grey Literature of Policy and Practice Recommendations on CALD Engagement in Clinical Trials**

| **Document (Organization, Year)** | **Primary Purpose** | **Relevance to Review** | **Key Messages Relevant to Equitable Clinical Trial Engagement, Involvement, and Participation** |
| --- | --- | --- | --- |
| Clinical Trial Awareness and Access amongst CALD Populations: Environmental Scan (ACTA; 2020) | Environmental scan: Examine barriers and current practices influencing CALD access to Australian clinical trials | Identifies baseline evidence regarding barriers experienced by CALD communities and organisational gaps | Limited awareness of clinical trials among CALD communities; Clinician gatekeeping; Language and interpreter barriers; Limited CALD data collection; Need for culturally responsive recruitment and research practices |
| Advancing Clinical Trial Engagement, Involvement and Participation for People from CALD Backgrounds (ACTA; 2023) | National recommendations: Provide consensus recommendations to improve equitable clinical trial engagement | Comprehensive national recommendations spanning policy, workforce, infrastructure, and community engagement | Community partnerships; Community research literacy; Workforce capability; Interpreter access; Multilingual resources; Routine CALD data collection; Consumer involvement; National coordination and accountability |
| National Clinical Trials Governance Framework and User Guide (Australian Commission on Safety and Quality in Health Care, 2022) | National governance framework: Establish governance standards for organisations conducting clinical trials | Positions equity, person-centredness, and consumer partnership as core quality requirements | Equity; Person-centred care; Consumer partnership; Health literacy; Cultural safety; Governance; Continuous quality improvement |
| National Standard Operating Procedures (SOPs) for Clinical Trials (National Clinical Trials Project; 2020) | National operational guidance: Standardise operational conduct of Australian clinical trials | Operational guidance supporting consistent participant-centred trial conduct | Standardised trial conduct; Participant communication; Documentation; Quality management; Consistency across trial sites. |
| National Health and Medical Research Strategy 2026–2036 (Australian Government, 2026) | National strategy: Define Australia's future health and medical research priorities | Reinforces national commitment to equitable research participation and translation | Inclusive research; Consumer and community involvement; Health equity; National research capacity; Translation into practice |
| National Safety and Quality Health Service Standards (2nd ed.) (Australian Commission on Safety and Quality in Health Care, 2021) | National health service standards: Establish national standards for safe, high-quality healthcare | Provides broader healthcare expectations that support inclusive research practice | Partnering with consumers; Health literacy; Diversity-responsive communication; Consumer governance; Responding to diverse consumer needs |
| National Statement on Ethical Conduct in Human Research (NHMRC, ARC & Universities Australia, 2023) | National ethical guideline: Establish ethical principles governing human research | Provides ethical foundation underpinning equitable inclusion in research | Justice; Respect; Beneficence; Research merit and integrity; Informed consent; Fair participant selection |
